# Impact and Failure Analysis of U-Shaped Concrete Containing Polyurethane Materials: Deep Learning and Digital Imaging Correlation-Based Approach

**DOI:** 10.3390/polym17091245

**Published:** 2025-05-02

**Authors:** Saleh Ahmad Laqsum, Han Zhu, Sadi I. Haruna, Yasser E. Ibrahim, Mohammed Amer, Ali Al-Shawafi, Omar Shabbir Ahmed

**Affiliations:** 1 School of Civil Engineering, Tianjin University, Tianjin 300350, China; salehahmedlaqsum@gmail.com (S.A.L.); amer@tju.edu.cn (M.A.); ali91@tju.edu.cn (A.A.-S.); 2 Key Laboratory of Coast Civil Structure Safety of the Ministry of Education, Tianjin University, Tianjin 300350, China; 3 Engineering Management Department, College of Engineering, Prince Sultan University, Riyadh 11586, Saudi Arabia; ymansour@psu.edu.sa (Y.E.I.); oahmed@psu.edu.sa (O.S.A.)

**Keywords:** concrete, polyurethane, convolutional neural networks, U-shaped specimen, impact resistance

## Abstract

This study investigates the use of advanced convolutional neural networks (CNNs) to analyze and classify the fracture behavior of U-shaped concrete modified with polyurethane (PU) under repeated drop-weight impact loads. A total of 17 U-shaped specimens were tested under multiple drop-weight impact loads for each PU binder content (0%, 10%, 20%, and 30%) by weight of cement. By integrating digital image correlation (DIC) with dynamic and static mechanical testing, this research evaluates the concrete’s impact resistance and flexural behavior with varying PU binder content. Three CNN architectures, InceptionV3, MobileNet, and DenseNet121, were trained on a dataset comprising 1655 high-resolution crack images to classify the failure stages into no crack, initial crack, and advanced failure. Experimental results revealed that 20% PU content optimally enhances impact resistance and flexural strength, while mechanical properties declined significantly with 30% PU content. The strain localization in DIC analysis indicated reduced matrix cohesion, which was measured by the extent of strain concentration in the material, highlighting the importance of maintaining PU content below 20% to avoid compromising structural integrity. Among the models, InceptionV3 demonstrated superior accuracy (96.67%), precision, and recall, outperforming MobileNet (94.56%) and DenseNet121 (90.03%). The combination of DIC and deep learning offers a robust, automated framework for crack assessment, significantly improving accuracy and efficiency over traditional methods such as visual inspections, which are time-consuming and reliant on expert judgment.

## 1. Introduction

Concrete is one of the most widely used construction materials in infrastructure and building projects due to its high strength and durability [1]. It is utilized in constructing bridges, buildings, and roads due to its cost-effectiveness and excellent mechanical properties. However, the performance of concrete can be significantly affected by harsh environmental conditions and when subjected to dynamic loads, resulting in structural cracks within stress regions [2,3,4]. Cracks, ranging from the microscale to the macroscale, serve as early indicators of structural distress. Their propagation weakens the concrete matrix and compromises the overall structural integrity [5]. Microscopic cracks already impact the material’s performance, and visible cracks further exacerbate structural vulnerabilities by facilitating moisture ingress, accelerating reinforcement corrosion, and reducing load-bearing capacity. To address these challenges, polymer-cement-based concrete has been widely adopted as a repair material in civil engineering applications [6,7]. Previous studies have demonstrated that PU binders enhance durability [8] and impact resistance [9], making them a promising alternative for improving concrete performance. PU, in particular, has shown potential as an effective material for enhancing the longevity and resilience of concrete structures [10]. The deterioration of civil engineering structures is attributed to multiple factors, including corrosion caused by harsh environmental exposure, deicing salts, alkali-induced degradation, and deficiencies in design or construction practices. These factors contribute to crack formation, which occurs progressively over multiple stages, ultimately affecting the structural integrity of concrete-based infrastructure [11,12]. Therefore, we are analyzing and classifying the fracture behavior of U-shaped concrete modified with polyurethane (PU) under repeated drop-weight impact loads through deep learning and DIC techniques, in addition to analyzing failure patterns using DIC technology and linking them to the deep learning model. Therefore, accurately identifying the initiation and location of the first crack in concrete structures is essential for assessing structural integrity [13,14]. The relationship between the first and final cracks aids in evaluating structural behavior under dynamic loads [9,15]. Wattanapanich et al. [16] conducted a critical research review aimed at providing novel methods for using recycled aggregate in concrete through machine learning models. Imjai et al. [17] developed a novel equation for estimating the shear strength of concrete involving recycled aggregate. Hemalatha et al. [18] used DIC and conventional methods to study the behavior of reinforced concrete (RC) beams incorporated with short PVA fibers under a flexural test. Li et al. [19] used a DIC system to measure the fracture process of self-compacting concrete under a 3-point bending test. Haruna et al. [20] studied the bond behavior of repaired concrete using polyurethane grouting materials using a statistical analysis approach.

Traditional inspection methods, such as visual examination, are commonly used but are error-prone, time-consuming, and reliant on expert judgment, making them less reliable and potentially hazardous [14,21,22]. To overcome these limitations, machine learning algorithms and automation-based techniques, such as drones, image recognition models like U-Net, computer vision systems, sensors, and strain gauges, are used. These methods have demonstrated high efficiency in damage assessment speed, cost-effectiveness, and the reduction in excessive workload and accidents [23,24,25]. Moreover, these techniques can be more objective and reliable as condition assessments are determined through computational algorithms [26]. Many studies have investigated the possibility of automated inspection of concrete structures via image processing techniques (IPTs) and deep learning methods. Zhou et al. [27] introduced an R-Unet model that combines edge detection and attention mechanisms to enhance concrete crack segmentation. This method achieved 95.75% accuracy and effectively addressed challenges in low-contrast crack detection. Koch et al. [2] reviewed computer vision techniques for detecting cracks and defects in concrete and asphalt infrastructure [28]. While advancements have improved automation, challenges such as noise sensitivity and handling complex geometries remain. While crack widths are estimated with an error margin below 11%, the method struggled to identify small cracks in blurred images [29]. However, edge detection methods to improve crack detection achieve higher accuracy and reduce false positives using the U-Net algorithm [30]. Palomino et al. [31] developed and evaluated CNN models, including MobileNet, DenseNet121, ResNet50, and VGG16, to classify failure types in concrete cylinders during compression testing. Using a proprietary dataset of 2650 images, the study demonstrated that MobileNet attained an accuracy of 96%, the highest recorded accuracy, outperforming other models. Islam et al. [32] used transfer learning with CNN models, achieving 99.90% accuracy in concrete crack detection. This high accuracy was achieved using a balanced dataset, which was augmented through data augmentation techniques to improve model generalization and prevent overfitting. Li et al. [33] developed an intelligent inspection terminal for road damage detection. A modified SSD-MobileNet model trained on a custom database achieved 80.87% average precision in detecting common road damage, including cracks, potholes, and rutting, demonstrating the method’s effectiveness and efficiency. Ye et al. [34] developed STCNet I, a deep learning model with dilated convolution for rapid crack detection in slab tracks. Using a custom database of 48,000 images, the model achieved 99.54% accuracy and improved efficiency by reducing computational parameters and processing time compared to traditional models.

This research employs CNNs to enhance PUMC crack classification using advanced deep learning models, including MobileNet, DenseNet121, and InceptionV3. This approach aims to improve diagnostic accuracy while reducing assessment costs by providing an innovative alternative to conventional crack evaluation methods. Utilizing the ACI 544-2R test procedure [35] enables more nuanced information extraction regarding structural failure mechanisms. The proposed methodology integrates advanced machine learning techniques with DIC technique protocols, establishing a robust, quantitative approach to concrete failure analysis. This innovative framework not only improves diagnostic accuracy but also introduces a more systematic methodology for understanding complex structural degradation processes and enables real-time crack evaluation, representing a significant advancement in infrastructure assessment techniques.

## 2. Materials and Methods

### 2.1. Materials

**Figure 1:**
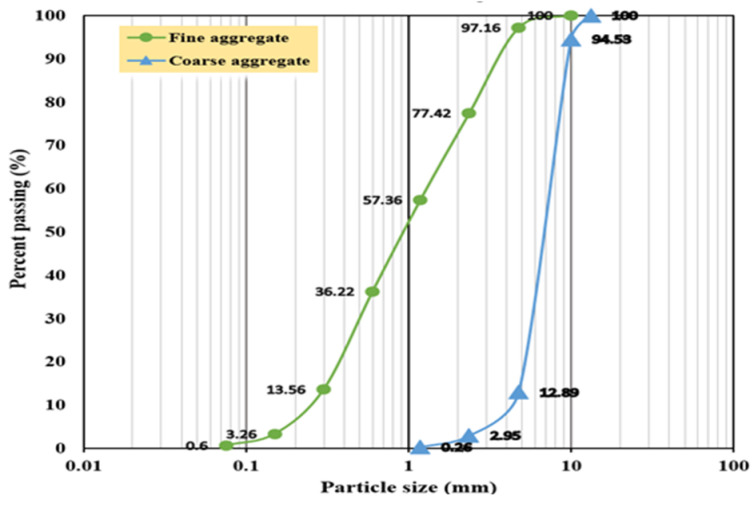
Gradation curve of aggregates used.

PUMC specimens were prepared to assess impact resistance properties using grade 42.5R cement [36]. The fine aggregate consisted of natural river sand with a maximum particle size of 4.75 mm, a fineness modulus of 2.82, and an apparent density of 2626 kg/m^3^. Coarse aggregates from crushed natural stone ranged in size from 5 to 16 mm, with an apparent density of 2600 kg/m^3^ and a fineness modulus of 2.67. The distribution of aggregate particle sizes is illustrated in Figure 1. To enhance the workability of PUMC mixtures, a polycarboxylate-based superplasticizer with a 20% water-reducing capability was incorporated at a dosage of 0.15% by weight of cement, which is consistent with previous studies [9,32].

#### 2.1.1. Polyurethane (PU) Structure

**Figure 2:**
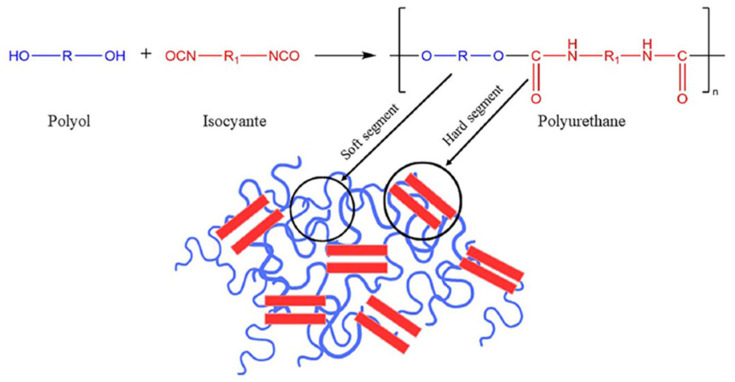
General structure of polyurethane.

PU is composed of a diol containing hydroxyl (-OH) groups and a di- or polyisocyanate (-NCO) group, linked through urethane bonds. The synthesis process involves an exothermic reaction between polyol and isocyanate [37,38], as shown in Figure 2. This study synthesized the PU binder by mixing castor oil containing CaCO_3_, as the main chemical composition (polyol), and polyaryl polymethylene isocyanate (PAPI) at a 6:1 mixing ratio (Table 1), following the procedure adopted in previous studies [15,39,40].

The mechanical properties of PU vary depending on the synthesis method. Its structure comprises hard segments from isocyanates, contributing to strength and crystallinity due to their high glass transition temperature (Tg, typically a temperature between 50 and 80 °C), and soft segments from polyols, which enhance flexibility due to their lower glass transition temperature (Tg, typically between temperature ranges of −50 °C and −10 °C). This contrast in Tg leads to microphase separation, a fundamental property that influences PU’s adaptability [41]. PU’s tensile characteristics can be tailored for specific applications. The key physical properties of the PU binder are summarized in Table 1.

#### 2.1.2. Mix Proportion and Specimen Preparation

**Figure 3:**
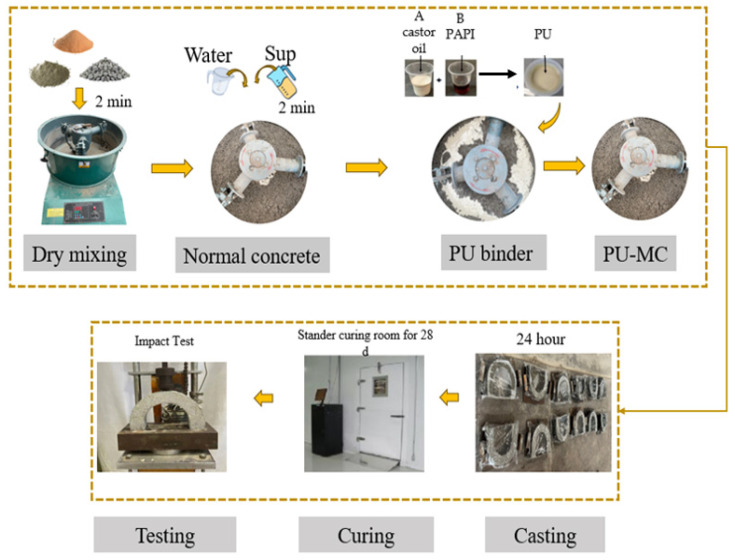
The schematic illustration for the preparation of PUMC.

Table 2 outlines the mixed proportions of concrete incorporating varying PU binder contents. The concrete mixture was designed to achieve a target compressive strength of 50 MPa. The PU binder was introduced into the concrete mixture at the following four levels: 0, 10, 20, and 30% by weight of cement. These PU binder contents were selected following previous research by Laqsum et al. [9], Alkahtani et al. [8], and Yilin et al. [42]. A fixed water-to-cement ratio of 0.4 was maintained for all PUMC mixtures. The preparation process involved placing the primary materials, sand, coarse aggregates, and cement into a concrete mixer for dry mixing over approximately two minutes. Subsequently, water and a superplasticizer were incorporated into the dry mix, followed by an additional three minutes of mixing. The freshly prepared PU binder was then added to the concrete mixture, and mixing continued until a uniform consistency was achieved. The PUMC mixtures were cast into U-shaped and cube molds for experimental testing and cured at room temperature for 24 h. Afterward, they were transferred to a controlled curing environment set at 20 ± 2 °C with a relative humidity of 98 ± 2% for 28 days before the test. A detailed schematic of the PUMC preparation process is illustrated in Figure 3.

### 2.2. Testing Method

#### 2.2.1. Drop-Weight Impact Test

**Figure 4:**
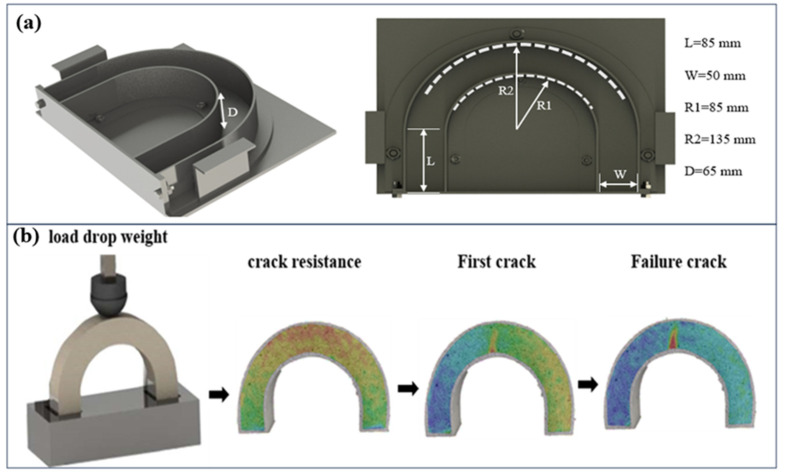
(**a**) U-shaped specimen dimensions. (**b**) Stages of cracks in the U-shaped specimen.

Figure 4a illustrates the U-shaped concrete specimen employed in this investigation. The impact resistance testing methodology closely aligns with established protocols from previous research [9,43,44,45]. Specifically, the experimental setup involved a 0.875 kg steel hammer head with a 60 mm diameter positioned at the midpoint of the U-shaped concrete specimen via a sliding rail transfer system. The experimental protocol focused on documenting two critical failure parameters. The first parameter, *N*_1_, represents the number of impact drops required to initiate the initial surface cracks. The second parameter, *N*_2_, quantifies the number of drops necessary to achieve complete specimen failure, indicated by the appearance of a dominant crack visible both through DIC and visual inspection. Notably, a preliminary stage designated *N*_0_ precedes crack formation, representing the pre-crack condition. The comprehensive crack pattern depicted in Figure 4b provides nuanced insight into the material degradation mechanisms. The crack classification was systematically implemented, distinguishing between initial microfractures *N*_1_ and catastrophic structural failure *N*_2_. This testing method evaluates concrete structural integrity by systematically documenting crack initiation, propagation, and ultimate failure, providing critical insights into material resilience.

**Figure 5:**
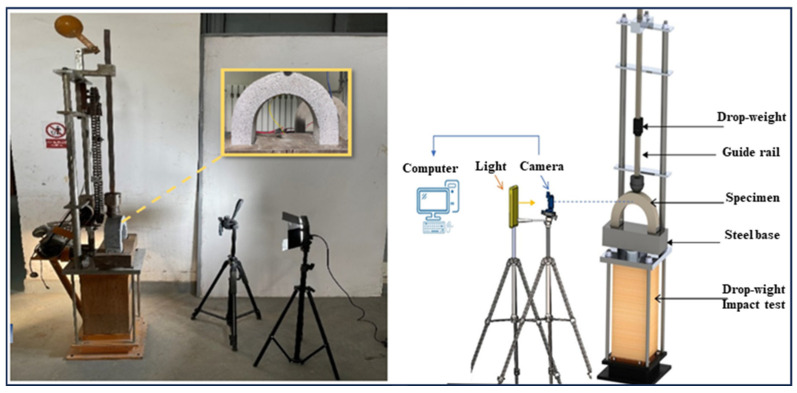
Drop-weight impact test configuration integrated with DIC system.

**Figure 6:**
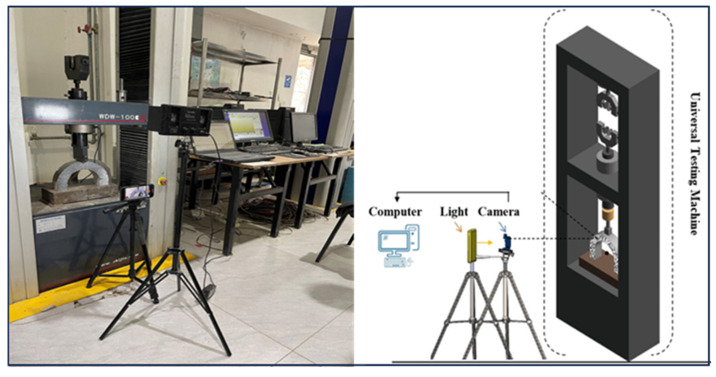
Static U-shaped flexural configuration integrated with DIC system.

We assessed the structural integrity of the specimens comprehensively to evaluate the structural integrity of the specimens. The first method involved a U-shaped drop-weight impact test utilizing the DIC technique (Figure 5), which was designed to assess the U-shaped specimens’ impact resistance and failure characteristics under dynamic loading conditions. The second approach consisted of a static flexural test of a U-shaped specimen incorporated with a DIC system (Figure 6), aimed at analyzing the mechanical response of the specimens under static loading. The flexural test using a U-shaped specimen was conducted following the Chinese standard GB/T 50081-81 [46]. During the test, the loading rate was maintained consistently at 0.05 MPa/s. The integration of these methodologies facilitated a systematic examination of crack initiation, propagation, and ultimate failure, providing a comprehensive assessment of the resilience of PUMC material under both dynamic and static loading conditions.

#### 2.2.2. Digital Image Correlation (DIC) Analysis

Figure 5 and Figure 6 show the experimental setup for the DIC analysis, which includes a high-resolution camera, a computer, and a controlled lighting system, all arranged to optimize image accuracy. U-shaped specimens underwent multiple drop-weight impact trials to evaluate failure mechanisms. Before testing, each specimen was cleaned, and a high-contrast white–black speckle pattern was applied to enhance image tracking and deformation analysis. The speckle pattern, consisting of randomly distributed black and white dots, uniformly covered the U-shaped region with particle sizes ranging from 1 to 1.5 mm. DIC calibration was performed using a 48 MP camera positioned at a fixed distance from the specimen, with a gauge length set at 50 mm, ƒ/1.78 aperture, second-generation sensor-shift optical image stabilization, a seven-element lens, and 100% focus pixels. The calibration included adjusting the camera focus and correcting lens distortion for accurate strain measurements. A neutral white LED spotlight ensured consistent image quality, minimizing ambient light variations. Both image processing and analysis were conducted on a high-performance computing system to track strain and crack propagation precisely.

### 2.3. Development of a Deep Learning Algorithm to Evaluate the Crack

**Figure 7:**
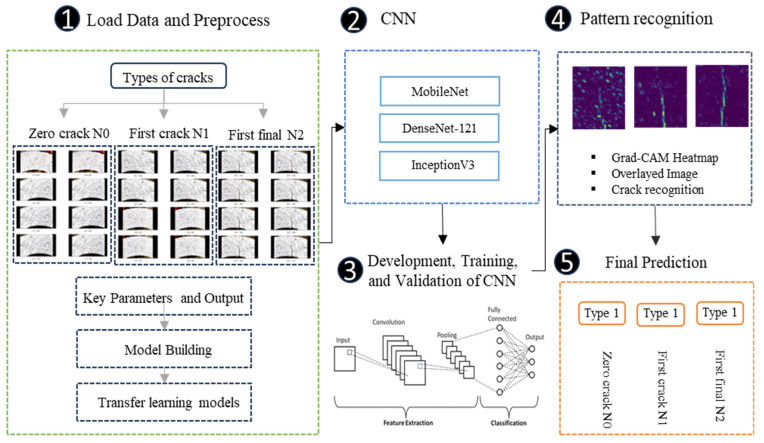
Flowchart of the proposed approach.

This study employed a deep learning approach to evaluate crack formation in U-shaped concrete samples to predict failure types in concrete. Figure 7 presents a structured flowchart summarizing this study. Failure images acquired through the DIC method were classified into three categories: no cracks *N*_0_, crack initiation *N*_1_, and crack propagation *N*_2_. A CNN was implemented in Python 3.10 to process and evaluate the dataset. The dataset was systematically partitioned into training, validation, and test sets to ensure reliable model performance assessment. Failure classification in the test set evaluated the model’s performance using well-established metrics, including accuracy, sensitivity, and specificity. The deep learning framework was developed using machine learning libraries such as TensorFlow and Keras, which facilitated model construction and optimization.

#### 2.3.1. Databases

**Figure 8:**
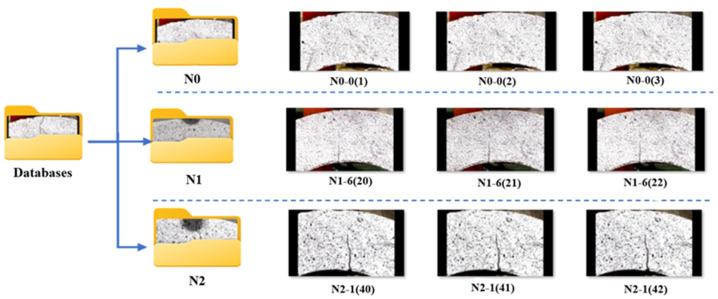
Methodology flowchart.

Figure 8 shows the concrete U-shaped specimen crack morphologies, with the geometrically designed U-shaped configuration facilitating controlled crack formation and propagation. A crack classification methodology delineated the following three types of cracks: type *N*_0_ (pre-crack initialization, 553 images), type *N*_1_ (initial crack development, 551 images), and type *N*_2_ (advanced structural failure 551 images). Therefore, the dataset encompassed a comprehensive collection of 1655 high-resolution images, each captured at 1920 × 1080 pixels utilizing standard RGB color channel representation. The dataset images were divided into three classes: *N*_0_ (553 images), *N*_1_ (551 images), and *N*_2_ (551 images). Stratified partitioning was applied to maintain class balance across splits, resulting in approximately 60% for training (933), 20% for validation (331), and 20% for testing (331).

To optimize computational efficiency and enhance machine learning model performance, images underwent a standardized preprocessing protocol. Specifically, the specimens were systematically cropped to a uniform 224 × 224 pixel dimension, meticulously accounting for variations in surface textures and illumination conditions. This resolution was consistently applied across all models, including InceptionV3, MobileNet, and DenseNet121, enabling a fair and balanced comparison of their classification processes [47]. Furthermore, stratified splitting was employed to ensure balanced *N*_0_, *N*_1_, and *N*_2_ class distributions across the training, validation, and test sets. This preprocessing approach demonstrated significant methodological advantages, notably reducing computational complexity and enabling predictive accuracy exceeding 90% [48,49]. The image dataset was meticulously organized and stored in a consolidated file structure, categorized according to precise crack typology, thus facilitating streamlined data management and analytical processing. Figure 8 depicts the study’s methodological framework.

#### 2.3.2. Conventional Neural Networks (CNNs)

**Figure 9:**
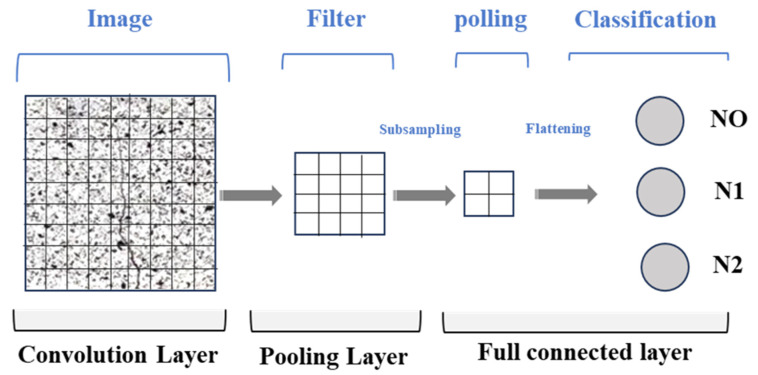
CNN structure.

CNNs represent an advanced machine learning methodology for complex pattern recognition and are particularly effective in computational image analysis and classification tasks [50]. Figure 9 shows the architecture of a CNN. It begins by transforming input images into discrete pixel value matrices, which are subsequently subjected to specialized convolutional filters designed to extract progressively abstract spatial features. These convolutional kernels perform multiple transformations at each layer. Pooling operations then reduce dimensional complexity, optimizing computational efficiency and mitigating potential computational overhead. The network architecture subsequently culminates in a critical dimensionality reduction phase, where multidimensional feature representations are systematically flattened into a linear vector, facilitating the final classification stage. In the terminal classification phase, machine learning algorithms probabilistically determine the specific concrete failure typology, translating complex visual information into meaningful, quantifiable structural insights. This technique leverages advanced computational methodologies to intelligently process and interpret visual data across multiple neural network transformation stages, demonstrating the powerful potential of deep learning in structural material analysis.

The computational framework was implemented using the Keras deep learning library in Python, offering a sophisticated neural network interface compatible with TensorFlow and other computational backends. Experimental data originating from impact testing were systematically preprocessed, with input parameters meticulously configured across multiple CNN architectures. The dataset underwent stratified partitioning of 60% training (933), 20% validation (331), and 20% testing (331). Specifically, for all models (MobileNet, DenseNet121, and InceptionV3), a batch size of 32, 40 epochs, a learning rate of 0.0001 (with the Adam optimizer), and a dropout rate of 0.5 were used. In addition, dropout regularization techniques were applied within the models to reduce overfitting and improve generalization during training.

#### 2.3.3. MobileNet

**Figure 10:**
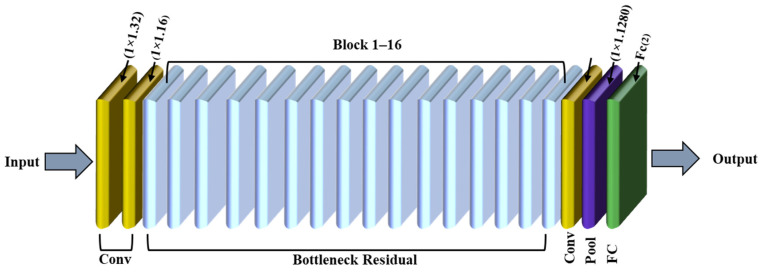
MobileNet model.

Developed by Google researchers, MobileNet represents an innovative CNN architecture designed to optimize computational efficiency without compromising performance metrics. The network’s sophisticated design incorporates strategically engineered components that enable advanced image processing and classification capabilities. Figure 10 illustrates the MobileNet architecture [51], which is designed for efficient image processing. It begins with an input layer that accepts 224 × 224 pixel images with three color channels. The data are then processed through depthwise separable convolutional layers, including convolutions, to extract features efficiently. Bottleneck residual layers further reduce the input dimensionality, optimizing computational requirements. An average pooling layer aggregates pixel clusters, followed by a fully connected layer with six neurons to classify concrete fault types based on the extracted features.

#### 2.3.4. DenseNet121

**Figure 11:**
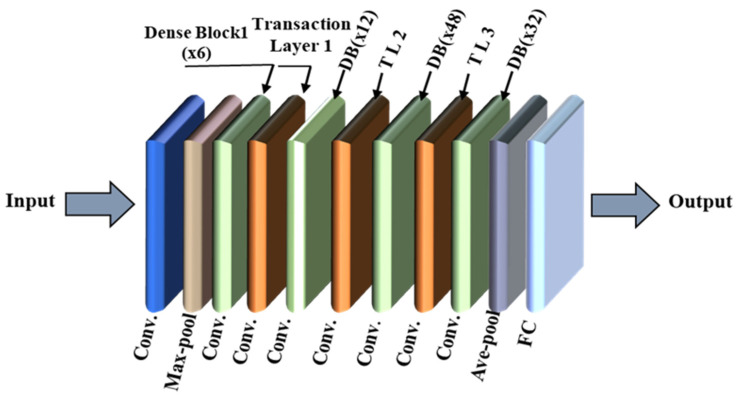
DenseNet121 model.

DenseNet121, part of the DenseNet family, is a deep CNN designed for efficient feature reuse and improved gradient flow through dense connectivity. Each layer receives input from all preceding layers, enabling better utilization of the extracted features. Figure 11 depicts the DenseNet121 architecture [52], starting with a convolutional layer (conv) to extract image features of concrete defects, followed by a max-pool to refine and reduce the initial features. The network includes four dense blocks, where each layer’s output is concatenated with the inputs to subsequent layers, enhancing feature sharing and information flow. Dense blocks and transition and pooling layers (ave-pool) reduce feature dimensions to control network size and computational complexity. Finally, fully connected layers classify the defect type based on the extracted features.

#### 2.3.5. InceptionV3

**Figure 12:**
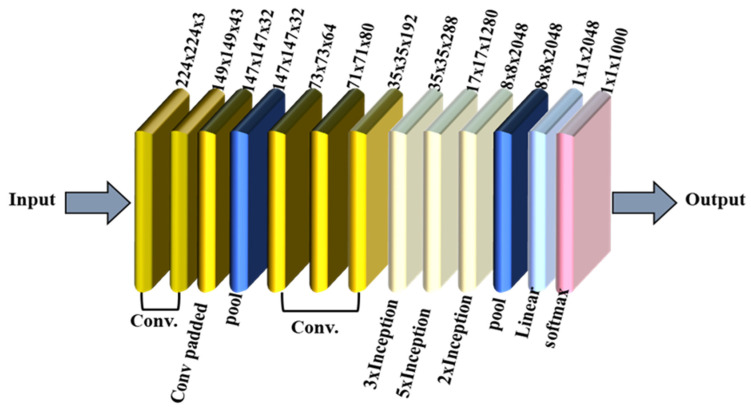
InceptionV3 model.

InceptionV3 is an advanced version of the Inception architecture developed by Google and optimized for image classification and object detection tasks. It builds on the success of its predecessors (InceptionV1 and InceptionV2) by improving computational efficiency and accuracy. Figure 12 depicts the InceptionV3 architecture [53]. The architecture begins with an input layer that processes 224 × 224 pixel RGB images, followed by convolutional layers to extract low-level features. The inception modules, which combine parallel operations such as 1 × 1, 3 × 3, and 5 × 5 convolutions and pooling to capture multiscale features, are the core of the design. Among these modules, reduction blocks downsample the data, balancing computational efficiency and feature retention. The final stages include a global average pooling layer for spatial feature aggregation and fully connected layers for classification, ensuring accurate and efficient image recognition.

#### 2.3.6. CNN Evaluation Method

The performance of the classification models in this study was systematically evaluated using key metrics, including accuracy, precision, recall, specificity, and F1-score, summarized in Table 3, which offer a comprehensive assessment of the model’s effectiveness across various performance dimensions. Accuracy measures the overall correctness of predictions, while precision and recall provide insights into the model’s ability to correctly identify positive instances and its sensitivity to detecting relevant patterns. Specificity evaluates the model’s capability to distinguish negative instances accurately, and the F1-score balances precision and recall. These metrics facilitate a holistic understanding of the model’s predictive capabilities and classification performance [54,55].

## 3. Results and Discussions

### 3.1. Impact Strength of U-Shaped PU-Modified Concrete

**Figure 13:**
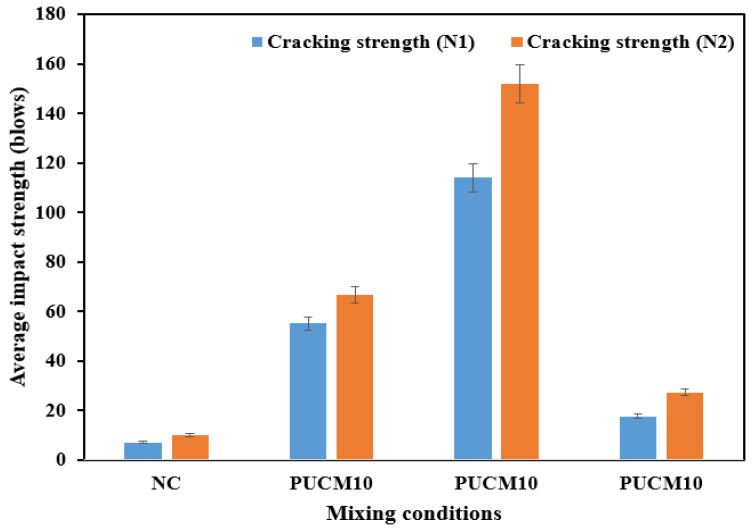
The average number of blows for crack initiation at the cracking stages.

Table 4 summarizes the impact resistance properties of an average of 17 U-shaped NC-PU specimens tested in each group using multiple drop-weight impact tests. The table highlights the average number of drops required to initiate the first visible crack, *N*_1_, and the number of drops leading to complete structural failure, *N*_2_, as illustrated in Figure 13. The results indicate that the impact resistance of polyurethane-modified concrete (PUMC10 and PUMC20) improved significantly due to incorporating PU binder at both cracking stages. However, a notable reduction in impact strength was observed in specimens with the highest PU content (30% PU). Upon the inclusion of the PU binder, the performance tends to decrease with increased polyurethane content (30% PU). The low performance is attributed to the adsorption of the cement particles, restraining hydration and generating more voids; hence, reducing the mechanical properties [56].

The average impact strength at both the initial cracking and complete failure stages increased with the addition of 10% and 20% PU binder content but decreased with 30% PU binder content (Table 4). The coefficient of variation (COV) for the seventeen specimens in each group showed that the highest COV value of 46% occurred at the initial crack stage (*N*_1_) of the control group, while the lowest COV of 21% was observed at the complete failure stage (*N*_2_) of the PUMC30 group. The PUMC10 group had COV values of 35% and 29% at the initial and complete failure stages, respectively, while PUMC20 had a COV of 39% at both stages. The larger error bars for PUMC20 reflect inherent variability at the optimal PU content (20%), likely due to threshold behavior in polyurethane–cement interactions, where minor fluctuations in PU dispersion, curing conditions, or bonding can amplify the variability in crack resistance.

Overall, comparing the results of this study with previous research using cylindrical specimens for repeated drop-weight impact tests, the COVs for the impact data were minimized due to the use of U-shaped specimens.

**Figure 14:**
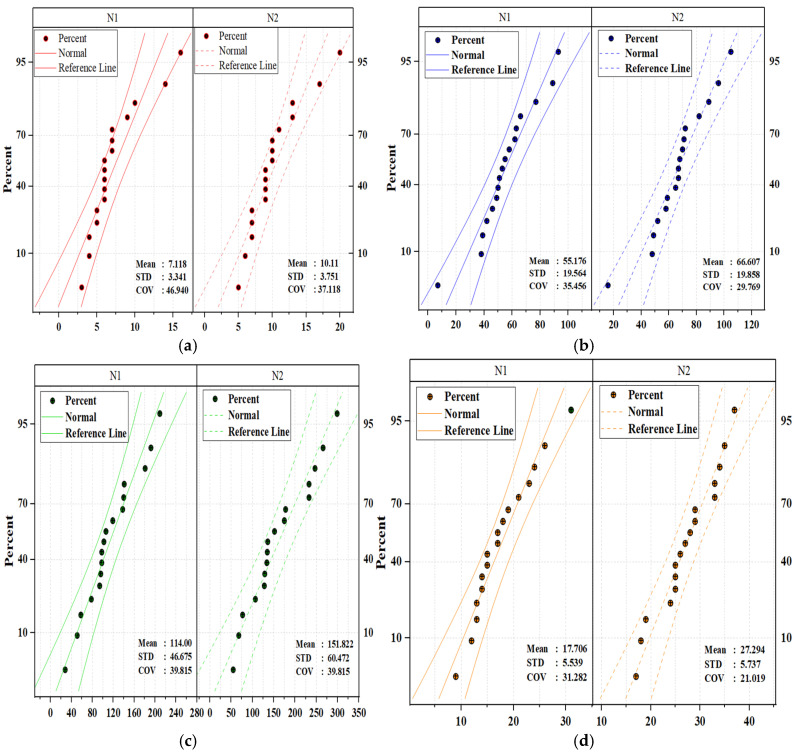
Normal probability plots of impact blow for (**a**) NC, (**b**) PUMC10, (**c**) PUMC20, and (**d**) PUMC30.

Figure 14 presents the normal probability distribution plots for impact resistance *N*_1_ and *N*_2_ in different PUMC specimens, illustrating the statistical dispersion and deviation from the reference normal curve while highlighting the effect of PU modification on impact resistance. The distributions of the data points for the specimens generally follow a normal distribution, with PUMC20 showing the tightest alignment to the reference normal curve, indicating a more consistent impact resistance performance, as seen in Figure 14c. The distributions for NC and PUMC10 are also normal-like but show slight deviations, as shown in Figure 14a,b. At the same time, PUMC30 displays a broader spread of datum points, indicating more variability in the results and a less predictable performance, as shown in Figure 14d.

The mean values for first crack initiation (*N*_1_) are 7.118 for NC, 55.176 for PUMC10, 114.0 for PUMC20, and 17.706 for PUMC30, while the mean values for complete failure (*N*_2_) are 10.118 for NC, 66.706 for PUMC10, 151.882 for PUMC20, and 27.294 for PUMC30, as shown in Table 4. A significant rightward shift in mean values for PUMC10 and PUMC20 indicates a higher number of impact blows required for both first crack initiation and complete failure, confirming the enhanced impact resistance resulting from PU modification. The coefficient of variation (COV) also demonstrates a trend of increasing reliability and consistency with PU incorporation. The PUMC20 specimen exhibits COV values of 40.943% for *N*_1_ and 39.815% for *N*_2_, signifying the most stable and predictable impact performance among all specimens. However, the PUMC30 specimen, while still outperforming NC (COV = 46.940% for *N*_1_, and 37.118% for *N*_2_), shows a smaller deviation in impact resistance values, with COV = 31.282% for *N*_1_ and 21.019% for *N*_2_, reflecting diminished improvements beyond the optimal PU content. Although the PUMC30 mix retains some benefits over standard concrete, it does not achieve the same toughness and energy absorption level as PUMC20, suggesting that excessive PU content beyond 20% leads to a decline in structural enhancement.

**Figure 15:**
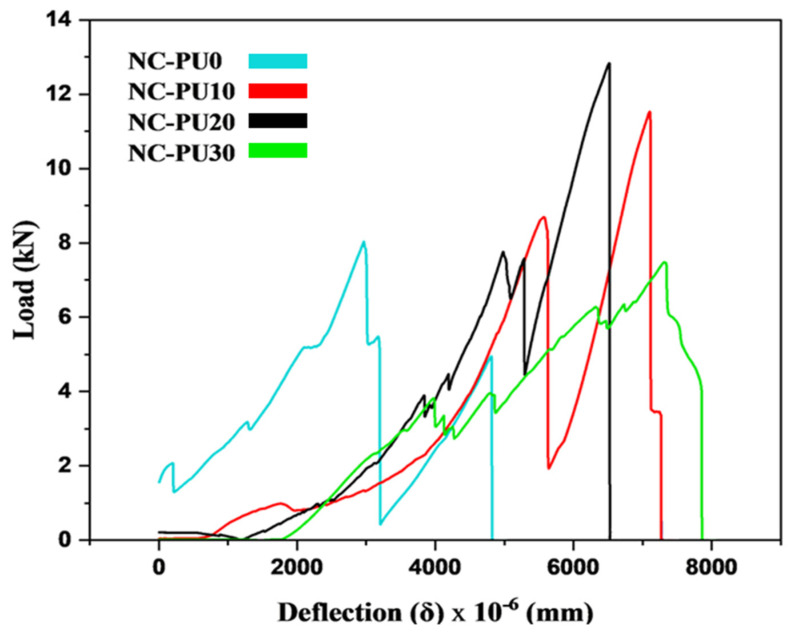
U-shaped static-load flexural test containing different PU content.

Figure 15 presents load versus deflection curves from static-load flexural tests following ASTM C78, conducted on concrete specimens modified with varying PU content (0%, 10%, 20%, and 30%). The results show that increasing PU content significantly affects the specimens’ load-bearing capacity and deformation characteristics. Specimens with 20% PU exhibit the highest secondary peak load capacity, followed by those with 10% and 30% PU. The results obtained in this study agree with findings from past studies [8,45]. However, flexural deformation differed due to the adoption of U-shaped specimens in this study instead of beam specimens. The first peak load does not represent the intrinsic flexural strength of the undamaged material. Due to the nature of the U-shaped specimen, the test specimen suddenly failed under flexural load in the case of the control. The secondary peak in the 20% PU specimens occurs after an initial load, reflecting post-crack toughness due to polymer chain entanglement and microcrack closure. After the first crack, the test specimen continued to carry load due to the viscoelastic properties of polyurethane materials. The gradual failure behavior of the PU-modified concrete (PUMC) specimens indicates enhanced energy dissipation capabilities, while the 30% PU specimens show reduced performance, with a lower peak load and brittle failure. The PUMC specimens (PUMC10 and PUMC20) exhibit quasi-ductile failure, with multiple microcracks forming before coalescing into a dominant crack, demonstrating better stress redistribution.

### 3.2. Failure Progression in U-Shaped Specimens Under Drop-Weight Using DIC

**Figure 16:**
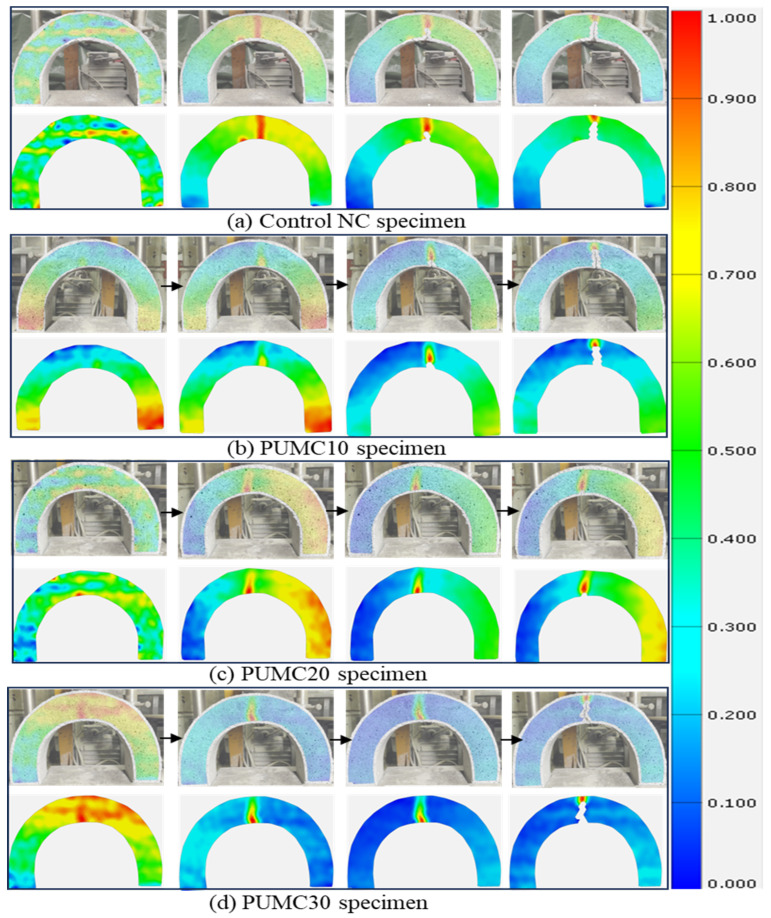
Failure progression of NC and PUMC using DIC technique under drop-weight.

**Figure 17:**
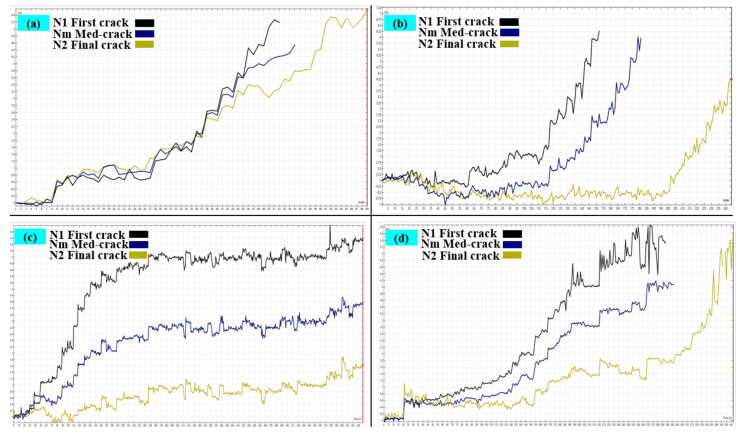
The cracking behavior under dynamic loads: (**a**) NC, (**b**) PUMC10, (**c**) PUMC20, and (**d**) PUMC30.

Structural health monitoring has been a concern and a developing field of study. Real-time monitoring of building structure dependability presents a chance to reduce maintenance and inspection expenses while enhancing public safety [57,58,59]. DIC is a non-contact full-field testing method that monitors the failure of concrete materials. The DIC results revealed distinct failure mechanisms among the PUMC U-shaped specimens subjected to drop-weight impact loading. Figure 16 illustrates the progressive failure stages captured via DIC imaging. The top row presents the real-time crack evolution, while the bottom row displays the corresponding strain maps, highlighting stress concentration zones. The color transition from blue to red signifies increasing strain intensity, revealing critical failure points where stress accumulates beyond the material’s capacity. The failure pattern varies among specimens based on PU content. In the NC specimen (Figure 16a), a single dominant vertical crack suddenly forms at the bottom mid-span, extending directly toward the top surface, leading to an abrupt and catastrophic fracture with minimal energy dissipation. PUMC30 exhibits a large crack width (1.278 mm) compared to NC (1.236 mm) which arises from excessive PU content destabilizing the concrete matrix. At 30% PU, incomplete cement hydration and phase separation create voids and weak interfacial zones (Section 2.1.1), reducing cohesion. Unlike the ductile microcracking in PUMC20, strain localization in PUMC30 (Figure 16d) accelerates crack propagation due to poor stress redistribution. This aligns with studies showing that polymer–cement composites degrade mechanically beyond optimal polymer levels [8,36] as excessive PU disrupts the matrix, prioritizing brittleness over toughness. This behavior indicates a brittle failure mechanism, where stress is rapidly accumulated and released, as illustrated in Figure 17a. Conversely, Figure 17b,c illustrates that the PUMC10 and PUMC20 specimens exhibit a delayed failure progression, where multiple microcracks appear before the primary crack fully develops. The strain maps indicate a more even stress distribution, preventing sudden catastrophic failure, as shown in Figure 17.

Among these, the PUMC20 specimen demonstrates the highest strain energy absorption, as evidenced by a gradual increase in strain intensity over a larger area, further delaying fracture initiation. This suggests that PU incorporation enhances ductility, enabling the material to withstand greater impact loads before reaching a critical failure state. Additionally, crack propagation in PUMC specimens appears more controlled, often following a branching or stepwise pattern, in contrast to the sudden, linear fracture observed in NC specimens. From Figure 16d, the PUMC30 specimen presents a mixed failure pattern. While PU initially delays crack formation, the strain localizes more intensely once failure initiates, leading to a wider crack opening at the bend section. This suggests that excessive PU content compromises the concrete matrix under impact loads. Figure 16 also shows that the strain maps of PUCM10 and PUCM30 are relatively symmetric, whereas PUCM20 appears asymmetric. This asymmetry is attributed to the enhanced ductility at 20% PU content, which causes more distributed microcracking, as shown in Figure 17. In contrast, PUCM10 and PUCM30 exhibited more uniform crack paths and symmetric failure behavior.

**Figure 18:**
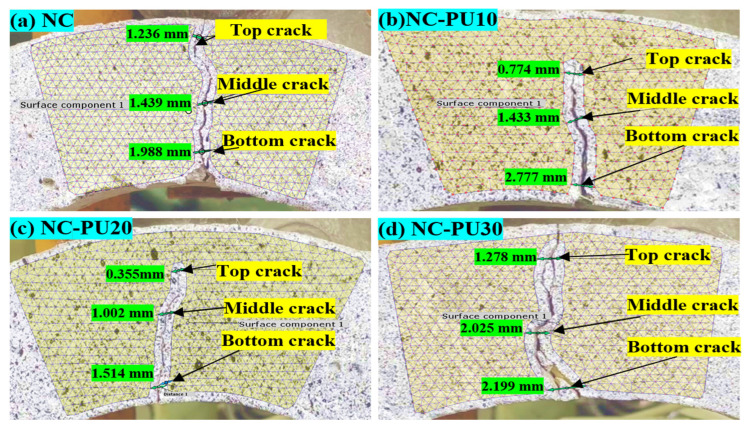
Failure development of crack width with DIC technique under impact load: (**a**) NC, (**b**) PUMC10, (**c**) PUMC20, and (**d**) PUMC30.

The crack width measurements using DIC in Figure 18 provide a quantitative assessment of fracture behavior under drop-weight impact, focusing on top crack width as a key indicator of crack development. The NC specimen exhibits the widest top crack at 1.236 mm, indicating rapid and uncontrolled crack propagation due to its brittle nature. In contrast, the PUCM10 specimen shows a reduced crack width of 0.774 mm, suggesting moderate improvement in crack resistance. The PUCM20 specimen demonstrates the smallest top crack width at 0.355 mm, confirming its enhanced ability to delay crack initiation and propagation. However, the PUCM30 specimen shows an increased crack width of 1.278 mm, exceeding both PUCM10 and PUCM20, indicating that excessive PU content may reduce crack control under impact. These results highlight the effectiveness of DIC technology in delivering accurate measurements while minimizing visual bias caused by surface irregularities.

### 3.3. Failure Progression in U-Shaped Specimens Under Static Load Using DIC

**Figure 19:**
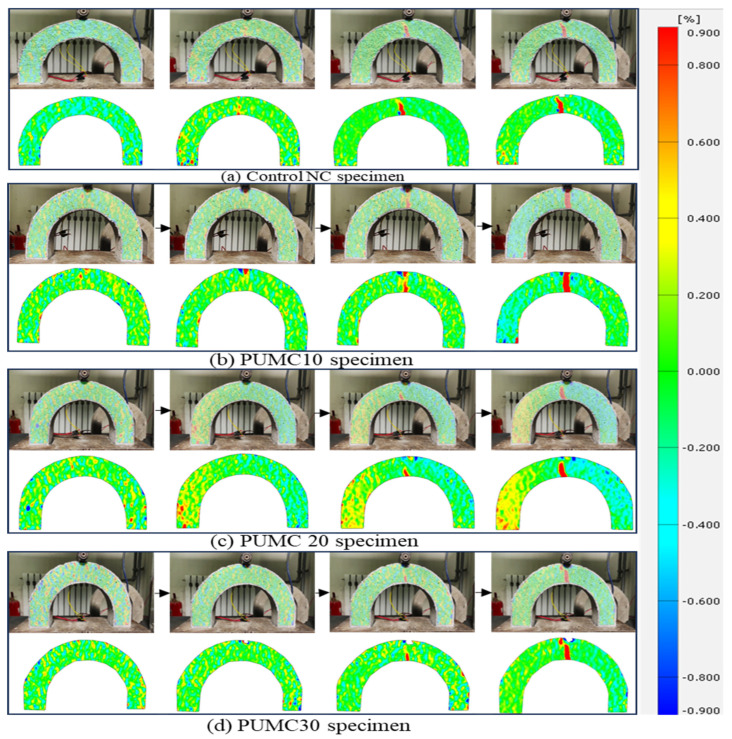
Failure progression of NC and PUMC using DIC technique under static load: (**a**) NC, (**b**) PUMC10, (**c**) PUMC20, and (**d**) PUMC30.

**Figure 20:**
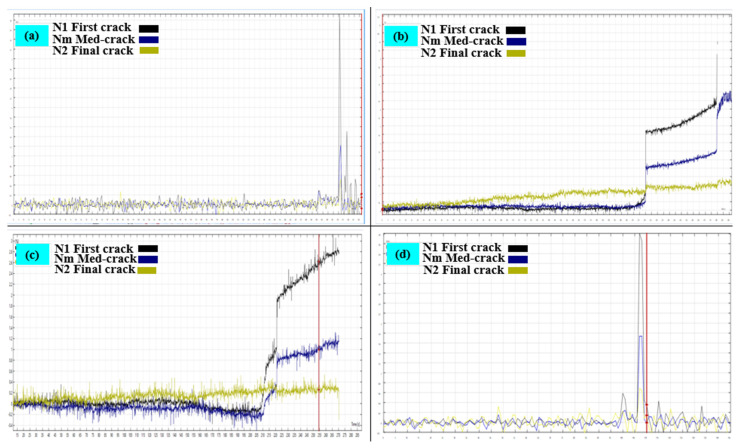
The cracking behavior under static load for (**a**) NC, (**b**) PUMC10, (**c**) PUMC20, and (**d**) PUMC30.

Figure 19 and Figure 20 illustrate the failure progression of U-shaped concrete specimens under static loading conditions analyzed using DIC. The control specimen (Figure 18 and Figure 19a) exhibits brittle failure, characterized by the abrupt formation of a single vertical crack at the mid-span, clearly indicated by a sharp transition to a red strain zone (high localized strain) against a predominantly blue background (low strain). This highlights rapid stress accumulation and minimal energy dissipation.

In contrast, the PUMC specimens show significantly improved ductility. The PUMC10 specimen (Figure 19 and Figure 20b) demonstrates more evenly distributed strain patterns before crack formation, highlighting improved stress redistribution and gradual microcracking. The PUMC20 specimen (Figure 19c) achieves optimal performance, with DIC strain maps indicating gradual color transitions, suggesting extensive strain distribution and delayed crack initiation. These patterns correspond to widespread microcracking and higher energy absorption capabilities, as shown in Figure 20c. The PUMC30 specimen (Figure 19 and Figure 20d) exhibits mixed behavior, initially resembling the strain distribution of PUMC20. However, following initial cracking, strain becomes intensely localized (bright red), resulting in rapid crack widening. This suggests that excessive PU content (30%) weakens the matrix cohesion and reduces structural integrity despite initially providing ductility benefits.

Overall, these results emphasize that PUMC enhances static flexural performance and dynamic impact resistance, especially at an optimal content of 20%. The controlled crack formation documented through DIC analysis further validates the effectiveness of the U-shaped specimen geometry in yielding consistent and reproducible test results, effectively reducing the data scatter typically encountered with conventional cylindrical specimens in impact testing.

## 4. Result of the Deep Learning Algorithm to Evaluate the Crack

This study evaluated the performance of three models, InceptionV3, MobileNet, and DenseNet121, for fracture image classification using a comprehensive dataset of 1650 images. These fracture images, obtained through experimentation using the DIC technique, were analyzed and compared to assess the accuracy of the models. Through a training and validation protocol, the study rigorously evaluated each model’s classification capabilities and predictive accuracy, providing a nuanced comparative analysis of their performance in fracture image recognition and characterization.

### 4.1. Performance Evaluation

**Figure 21:**
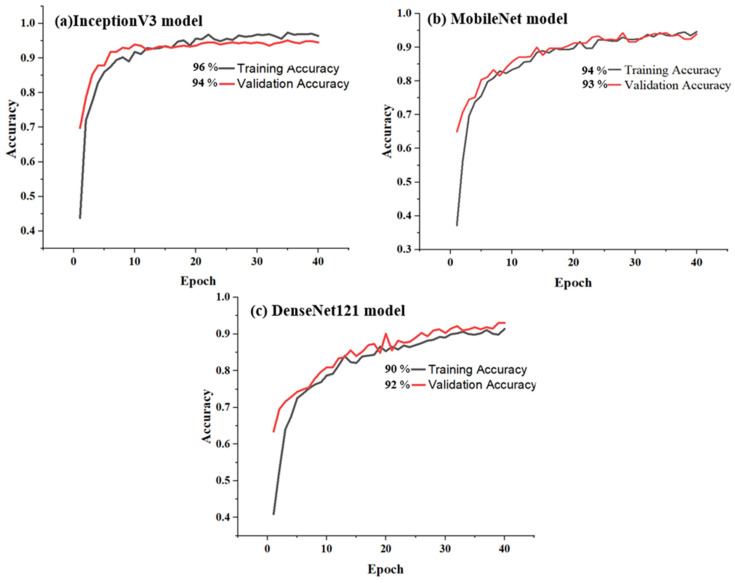
Accuracy of training and validation for models: (**a**) InceptionV3, (**b**) MobileNet, and (**c**) DenseNet121.

The results revealed nuanced performance characteristics across the three neural network architectures. The InceptionV3 model demonstrated superior classification capabilities, achieving 96% accuracy during training with a corresponding 4% error rate and maintaining 94% accuracy during validation with minimal performance degradation, as shown in Figure 21a. MobileNet exhibited comparably strong performance, registering 94% training and 93% validation accuracy with error rates ranging from 6% to 7%, as depicted in Figure 21b. The DenseNet121 model, while marginally less effective, still demonstrated robust classification capabilities, with 90% training accuracy and 92% validation accuracy, as illustrated in Figure 21c. Despite relatively small performance differences, the ability of the models to accurately classify fracture images across both training and validation stages highlights the potential of advanced deep learning techniques in medical image analysis. The minimal variance between training and validation results suggests the generalizability and reliability of the models in handling complex image classification tasks.

Table 5 presents the performance statistics of the InceptionV3 model in classifying three categories (*N*_0_, *N*_1_, and *N*_2_) based on precision (P), recall (R), and F1-score. *N*_0_ achieves a precision of 94.87%, a recall of 99.10%, and an F1-score of 96.94%, demonstrating excellent recall and overall balanced performance. *N*_1_ achieves a precision of 96.47%, a recall of 92.13%, and an F1-score of 94.25%, reflecting strong precision but slightly lower recall. *N*_2_ achieves the highest precision (98.44%), a recall of 97.69%, and an F1-score of 98.06%, indicating superior classification performance. Overall, the model attains an accuracy of 96.67%. The macro-average precision, recall, and F1-score are 96.59%, 96.31%, and 96.42%, respectively, while the weighted averages are 96.70%, 96.67%, and 96.66%, respectively. These metrics highlight the model’s exceptional and well-balanced classification performance across all categories.

Table 6 presents the performance statistics of the MobileNet model in classifying three categories (*N*_0_, *N*_1_, and *N*_2_) based on precision (P), recall (R), and F1-score. *N*_0_ achieves a precision of 90.98%, a recall of 99.10%, and an F1-score of 94.87%, demonstrating high recall with balanced performance across all metrics. *N*_1_ achieves a precision of 93.90%, a recall of 86.51%, and an F1-score of 90.05%, indicating intense precision but slightly lower recall. *N*_2_ achieves the highest precision (98.42%), a recall of 96.15%, and an F1-score of 97.27%, reflecting the most robust classification performance among the categories. Overall, the model attains an accuracy of 94.56%. The macro-average precision, recall, and F1-score are 94.43%, 93.92%, and 94.06%, respectively, while the weighted averages are 94.69%, 94.56%, and 94.52%, highlighting the model’s balanced performance across all categories.

Table 7 presents the performance statistics of the DenseNet121 model in classifying three categories (*N*_0_, *N*_1_, and *N*_2_) based on precision (P), recall (R), and F1-score. *N*_0_ achieves a precision of 86.4%, a recall of 96.42%, and an F1-score of 91.13%, indicating high recall but slightly lower precision. *N*_1_ achieves a precision of 84.33%, a recall of 78.65%, and an F1-score of 81.39%, reflecting relatively lower performance than the other categories. *N*_2_ achieves the highest precision (97.56%), a recall of 92.30%, and an F1-score of 94.86%, indicating strong classification performance. Overall, the model achieves an accuracy of 90.03%. The macro-average precision, recall, and F1-score are 89.43%, 89.12%, and 89.13%, respectively, while the weighted averages are 90.22%, 90.03%, and 89.98%. These metrics highlight the model’s solid performance, with room for improvement in handling certain categories.

### 4.2. Confusion Matrix Analysis

**Figure 22:**
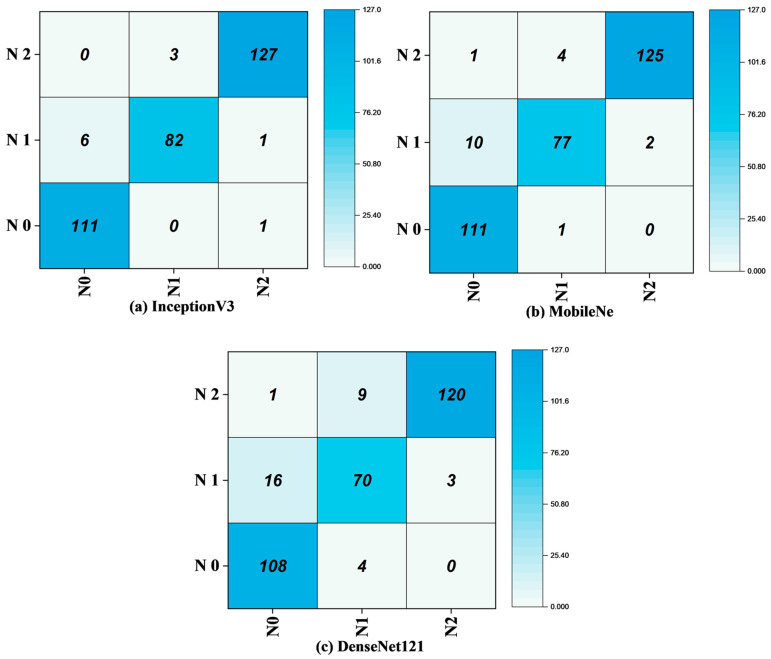
Confusion matrix of the models: (**a**) InceptionV3, (**b**) MobileNet, and (**c**) DenseNet121.

The confusion matrix is a performance evaluation tool that compares the actual class labels of test samples with the predicted class labels produced by the model. The diagonal elements of the matrix represent the correctly classified samples, while the off-diagonal elements highlight the misclassified samples. Figure 22 presents the confusion matrices for the InceptionV3, MobileNetV3, and DenseNet121 models. The InceptionV3 model demonstrates exceptional performance, with an overall accuracy of 96.67%. It shows strong classification results for *N*_2_, with 127 true positives, and has minimal misclassifications for *N*_0_ (six misclassifications) and *N*_1_ (three misclassifications). The false positive (FP) rate is low, with only a few instances of *N*_2_ misclassified as *N*_1_, and the false negatives (FN) are minimal, as seen in Figure 22a. This model’s ability to accurately identify fracture types highlights its superior pattern recognition and classification capabilities.

MobileNetV3, with an accuracy of 94%, exhibits good performance but has slightly higher misclassification rates compared to InceptionV3. The confusion matrix for MobileNetV3 (Figure 22b) shows that while it performs well in classifying *N*_0_ (111 true positives) and *N*_2_ (125 true positives), there is a noticeable drop in recall for *N*_1_, with 10 misclassified instances. Despite this, MobileNetV3 maintains solid performance across categories.

The DenseNet121 model, achieving an accuracy of 90.03%, shows robust classification abilities, particularly for *N*_2_ (120 true positives), as shown in Figure 21c. However, it has higher misclassification rates for *N*_0_ and *N*_1_, as shown in Figure 21c, where 108 instances of *N*_0_ are misclassified, and 16 instances of *N*_1_ are incorrectly classified. The false positive rate for *N*_0_ is notably higher, indicating that the model sometimes confuses the absence of cracks (*N*_0_) with the presence of minor cracks (*N*_1_). False negatives for *N*_1_ are also present, reflecting the model’s difficulty in detecting early crack stages. While DenseNet121’s performance is slightly lower than InceptionV3’s, it still provides valuable insights, particularly in identifying *N*_2_s, demonstrating the model’s potential in handling complex image classification tasks.

### 4.3. ROC Curve Analysis

**Figure 23:**
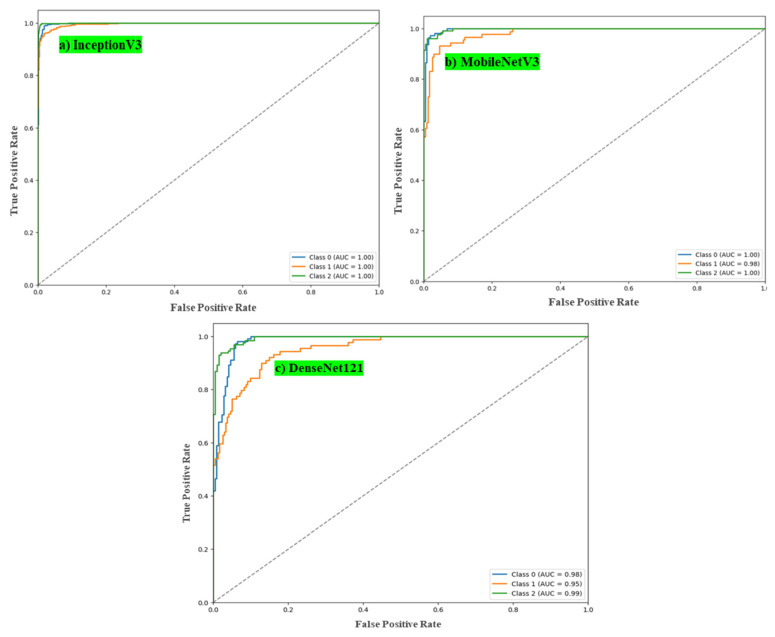
ROC for models.

The ROC curves are shown in Figure 23a,b. These demonstrate the classification performance of the InceptionV3 and MobileNet models. The MobileNet model achieved AUC values of 1.00 for Class 0, 0.98 for Class 1, and 1.00 for Class 2, indicating excellent class discrimination. Similarly, the InceptionV3 model exhibited outstanding ROC performance, with an AUC of 1.00 for all three classes. In contrast, the DenseNet121 model had AUC values of 0.98 for Class 0, 0.96 for Class 1, and 0.99 for Class 2, as shown in Figure 23c. Comparisons between the performances of these three CNNs are shown in Table 7.

The analysis of the results presented in Table 6 and Table 7 highlights that InceptionV3 outperforms the other models across all performance metrics. While DenseNet121 and MobileNet demonstrated strong and competitive performance, InceptionV3 exhibited superior capabilities in key areas. One notable challenge arises when *N*_1_ images (representing initial crack formation) visually overlap with *N*_0_ images (no cracks) or *N*_2_ images (advanced cracks). In such cases, fine cracks may be misclassified as part of the natural surface texture or incorrectly associated with the development of *N*_2_. This highlights the varying sensitivities of the models and subtle differences in crack morphology.

This comparative study, which evaluated the performance of InceptionV3, MobileNet, and DenseNet121 for detecting concrete deterioration underweight testing, underscores several advantages of InceptionV3. First, its superior accuracy in identifying crack propagation can be attributed to its ability to handle multiscale features effectively. The deeper architecture and the integration of auxiliary classifiers within InceptionV3 enhance feature extraction and classification, improving its detection performance. Second, InceptionV3 demonstrated higher precision, recall, and F1-scores in distinguishing between different stages of crack development. This ability allows for more accurate predictions of the progression of concrete deterioration. While all the models achieved commendable accuracy, stratification capabilities, and computational efficiency, InceptionV3 stands out as the most robust and reliable model for this specific application (Table 8).

## 5. Conclusions

This study systematically investigated the crack propagation behavior and structural performance of U-shaped PUMC under dynamic and static loading conditions, integrating advanced mechanical testing with deep learning-based image analysis. By employing DIC for strain mapping and training three CNN architectures—InceptionV3, MobileNet, and DenseNet121—on a dataset of 1655 high-resolution crack images, the research established a robust framework for automated crack classification and structural health assessment. The following key findings emerged:

1. PUMC20 specimens exhibited superior impact resistance, requiring an average of 151.9 blows to reach failure *N*_2_ compared to 27.3 blows for 30% PU and 10.1 for unmodified concrete. This optimal PU content improved energy absorption and delayed crack initiation. As captured by DIC, the enhanced strain distribution in PU20 specimens validated its efficacy in mitigating brittle failure.
2. In PUMC30, mechanical performance declined significantly. Crack widths increased to 1.278 mm under impact loading, exceeding even unmodified concrete (1.236 mm). The strain localization observed in DIC analysis indicated reduced matrix cohesion, highlighting the importance of maintaining PU content below 20% to avoid compromising structural integrity.
3. InceptionV3 outperformed other CNNs, achieving 96.67% accuracy, 98.44% precision for advanced cracks *N*_2_, and near-perfect AUC scores (1.00 for all classes). MobileNet and DenseNet121 demonstrated competitive but lower accuracies (94.56% and 90.03%, respectively), with DenseNet121 struggling to classify subtle *N*_1_ cracks (81.39% F1-score). The ability of the models to distinguish between pre-crack *N_0_*, initial crack *N*_1_, and advanced failure *N*_2_ stages underscores their potential for automating infrastructure inspections.
4. DIC analysis revealed stark contrasts in failure modes; unmodified concrete exhibited abrupt, single-crack propagation (brittle failure), while PUMC showed distributed microcracking and gradual strain evolution. PUMC specimens demonstrated the most uniform strain distribution.
5. The integration of DIC and CNNs effectively addresses key limitations of traditional inspection methods, such as subjectivity and time consumption. The proposed framework enables quantitative, real-time crack monitoring and classification through portable systems equipped with high-resolution imaging and lightweight CNN models deployed on edge devices. This offers a faster, more objective, and safer alternative for periodic inspections of structures such as bridges, sidewalks, and dams.
6. The current study is limited to seventeen U-shaped specimens from each group; the behavior of polyurethane can vary based on formulation and curing conditions, which are not fully accounted for, and the DIC captured surface deformations. Generally, this study used an idealized impact load test. It is therefore recommended for future studies to focus on testing more specimens with varying PU compositions, conducting parametric numerical simulations (FEAs), and microstructure analysis to validate the experimental result.

## Figures and Tables

**Table 1:** Indexes of PU binder.

PU Binder	Viscosity (CPS)	Appearance	Curing Time (h)		Tensile Strength(MPa)
			Initial	Final	
Castor oil	35,000	Gray-white and sticky	-	-	-
PAPI	250	Brown and transparent	-	-	-
PU binder	-	-	3.5	72	5.5

**Table 2:** Mix proportion of NC-PU mixtures (kg/m^3^).

Specimen ID	Cement	Sand	Coarse Aggregate	Water	PU Binder
NC-PU0	425	718	966	170	0.00
PUMC10	425	718	966	170	42.5
PUMC20	425	718	966	170	85.0
PUMC30	425	718	966	170	127.5

**Table 3:** Evaluation metrics for classification models.

Matric	Equation	Definition
Accuracy	$\frac{TP+TN}{TP+TN+FPFN}$	Accuracy refers to the ratio of correctly classified cases (both positive and negative) to the total number of cases.
Precision	$\frac{TP}{TP+FP}$	Measures the proportion of true positive predictions out of all instances predicted as positive, indicating how well the model avoids false positives.
Recall	$\frac{TP}{TP+FN}$	Measures the proportion of true positive cases that were correctly identified by the model.
F1-score	$\frac{2\times (precision+recall)}{precision+recall}$	The F1-score represents the harmonic mean of precision and recall. It is commonly used to assess a model’s balance between these two metrics.

**Table 4:** Impact resistance of the U-shaped NC-PU specimens (blows).

Parameter	NC		PUMC10		PUMC20		PUMC30	
	*N*_1_	*N*_2_	*N*_1_	*N*_2_	*N*_1_	*N*_2_	*N*_1_	*N*_2_
Mean	7	10	55	66	114	151	17	27
STD	3	3	19	19	46	60	5	5.7
COV (%)	46	37	35	29	40	39	31	21

**Table 5:** Performance metrics of the InceptionV3 model.

Type	Precision	Recall	F1-Score	Support	Model
*N*_0_	94.87	99.10	96.94	111	InceptionV3
*N*_1_	96.47	92.13	94.25	110	
*N*_2_	98.44	97.69	98.06	110	
accuracy	96.67	96.67	96.67	96	
macro-avg	96.59	96.31	96.42	331	
weighted avg	96.70	96.67	96.66	331	

**Table 6:** Performance metrics of MobileNet model.

Type	Precision	Recall	F1-Score	Support	Model
*N*_0_	90.98	99.10	94.87	111	MobileNet
*N*_1_	93.90	86.51	90.05	110	
*N*_2_	98.42	96.15	97.27	110	
Accuracy	94.56	94.56	94.56	94	
Macro-average	94.43	93.92	94.06	331	
Weighted average	94.69	94.56	94.52	331	

**Table 7:** Performance metrics of DenseNet121 model.

Type	Precision	Recall	F1-Score	Support	Model
*N*_0_	86.4	96.42	91.13	111	DenseNet121
*N*_1_	84.33	78.65	81.39	110	
*N*_2_	97.56	92.30	94.86	110	
accuracy	90.03	90.03	90.03	90	
macro-avg	89.43	89.12	89.13	331	
weighted avg	90.22	90.03	89.98	331	

**Table 8:** Comparison of ROC for models.

Class	ROC InceptionV3	ROC MobileNet	ROC DenseNet121
*N*_0_	1	1	98
*N*_1_	1	0.98	96
*N*_2_	1	1	99

## Data Availability

The original contributions presented in the study are included in the article. Further inquiries can be directed to the corresponding author.
